# Exploring Interventions to Improve the Oral Health and Related Health Behaviours of Adults Experiencing Severe and Multiple Disadvantage: Protocol for a Qualitative Study with Stakeholders

**DOI:** 10.3390/ijerph182211755

**Published:** 2021-11-09

**Authors:** Emma C. Joyes, Laura J. McGowan, Emma A. Adams, Martha Paisi, Martin Burrows, Hosein Shabaninejad, Fiona Beyer, Kate Haddow, Aishah Coyte, David Landes, Suzanne Moffatt, Richard G. Watt, Falko F. Sniehotta, Clare Bambra, Dawn Craig, Eileen Kaner, Sheena E. Ramsay

**Affiliations:** 1 Population Health Sciences Institute, Newcastle University, Newcastle upon Tyne NE2 4AX, UK; emma.joyes@newcastle.ac.uk (E.C.J.); emma.adams@newcastle.ac.uk (E.A.A.); Hosein.Shabaninejad@newcastle.ac.uk (H.S.); fiona.beyer@newcastle.ac.uk (F.B.); aishah.coyte@newcastle.ac.uk (A.C.); suzanne.moffatt@newcastle.ac.uk (S.M.); falko.sniehotta@newcastle.ac.uk (F.F.S.); Clare.bambra@newcastle.ac.uk (C.B.); dawn.craig@newcastle.ac.uk (D.C.); Eileen.kaner@newcastle.ac.uk (E.K.); sheena.ramsay@newcastle.ac.uk (S.E.R.); 2 Peninsula Dental School, University of Plymouth, Plymouth PL4 8AA, UK; martha.paisi@plymouth.ac.uk; 3 Inclusive Insight, Bournemouth BH6 5AY, UK; martin@inclusiveinsight.co.uk; 4 Fulfilling Lives Newcastle/Gateshead, Gateshead NE8 4DY, UK; kate.haddow@fulfillinglives-ng.org.uk; 5 Public Health England, Newcastle Upon Tyne NE15 8NY, UK; David.Landes@phe.gov.uk; 6 Department of Epidemiology and Public Health, University College London, London WC1E 7HB, UK; r.watt@ucl.ac.uk

**Keywords:** homelessness, multiple disadvantage, repeat offending, substance misuse, oral health, smoking, diet, qualitative study

## Abstract

The number of individuals in England experiencing homelessness, substance use, and involvement with the criminal justice system is increasing. These issues, referred to as severe and multiple disadvantage (SMD), are often interlinked and co-occur. Health inequalities, particularly poor oral health, persist for those facing these inter-related issues and are closely linked with high levels of substance use, smoking, and poor diet. However, evidence for interventions that can improve these health outcomes for those experiencing these issues is limited. This paper outlines the design of a qualitative study which aims to explore the perspectives of stakeholders to understand what interventions can help to support SMD groups with their oral health and related health behaviours (i.e., substance use, smoking, diet). Interviews and focus groups will be undertaken with stakeholders comprising two groups: (1) individuals with experience of SMD, and (2) service providers (staff and volunteers), policy makers, and commissioners who support such individuals. Public involvement and engagement is central to the project. For example, stakeholders and research partners in policy and practice and people with lived experience of SMD will provide input at all stages of this study. Findings from the study will inform an ‘evidence for practice’ briefing outlining recommendations for policy. Dissemination will occur through presentations to a range of practice, policy and academic beneficiaries, and through peer-reviewed publications.

## 1. Introduction

Individuals facing homelessness often experience substance misuse and involvement with the criminal justice system. These issues of homelessness, substance misuse and repeat offending often overlap and have been referred to as ‘severe and multiple disadvantage’ (SMD) [1]. There has been a large increase in the number of individuals experiencing SMD in England over the past decade [2], which occurred alongside prolonged austerity and cuts to local authority budgets. Furthermore, over two-thirds of people experiencing homelessness also report issues with substance use or involvement with the criminal justice system [1]. The criminal justice involvement of those who experience these inter-related disadvantages often relates to persistent, low-level offending, leading to community or short prison sentences [1]. Individuals receiving short prison sentences often experience issues related to substance misuse and will also re-offend when released into the community, indicating an unmet need which is not being addressed through prison sentencing [3]. Those leaving prison also often find themselves experiencing homelessness on release [1], indicating a cycle of difficulties leading to disadvantage.

These experiences of multiple disadvantage have major health impacts [1,4,5,6,7], including high levels of mental and physical ill-health [2,4]. Poor oral health is reported as one of the most common physical health problems faced by individuals experiencing SMD [4], with disproportionately high levels of tooth loss, untreated dental disease (caries/tooth decay, periodontal disease) and its complications, including infections and pain [8,9,10,11]. These complications often result in attendance at dental emergency clinics, or at accident and emergency departments [12]. Access to routine and preventive healthcare is extremely poor and non-attendance is common; poor attendance is also often related to the lived experience of homelessness, including disrupted lifestyles, not knowing how to access support, anxiety and social isolation [13,14]. Furthermore, the characteristics of the healthcare system (e.g., lack of training in engaging with socially excluded groups, the cost of care, and problems in registering patients with no fixed address) also serve as prominent barriers to dental care for individuals [14]. Poor oral health is also closely interlinked with health behaviours, particularly substance use, smoking, and high sugar consumption, which are also very common in people experiencing SMD [11].

Whilst issues related to oral health and health behaviours (e.g., substance use, smoking, and a poor diet) in SMD groups have been widely described, there is limited evidence on interventions that can address these health needs of SMD groups. In particular, evidence is limited on what would improve the implementation and acceptability of such interventions.

The aim of this qualitative study is to identify interventions that are acceptable and sustainable in improving the oral health and related health behaviours (substance misuse, smoking, diet) of adults facing SMD. In particular, this study will explore factors that influence the implementation of interventions (including settings, acceptability, resource implications, and potential adverse effects) of interventions to improve the oral health and related behaviours of adults experiencing SMD. This study will explore these issues from the perspective of people with experience of SMD, practitioners, service providers, and policy makers.

This study, along with a complementary systematic review, seeks to gather the evidence for effective and sustainable interventions to improve the oral health and related health behaviours of individuals experiencing SMD.

## 2. Materials and Methods

The qualitative research will explore solutions for oral health and related health behaviours from the perspective of two stakeholder groups through interviews and focus groups. The two groups are as follows: (1) individuals with experience of SMD, and (2) support service staff and volunteers, policy makers, and commissioners.

### 2.1. Inclusion Criteria

SMD population: Adults (aged 18 years and over) with experience of SMD (including homelessness, or more than one of homelessness, offending, substance misuse) in North East England (Newcastle/Gateshead areas) will be included. Homelessness will include a range of living situations, including those living in supported accommodation, insecure housing, sleeping on a friend/relative’s sofa (sofa surfing), and rough sleeping. The rationale for focusing on Newcastle/Gateshead is because this region has some of the highest levels of homelessness and drug-related deaths in England, as well as some of the highest levels of deprivation. The need for this research emerged from a local health needs assessment related to SMD populations in the Gateshead area, and this study seeks to find recommendations for local practice and policy for the populations of this region.

Service providers, commissioners, policy makers: Service providers, commissioners and policy makers who support individuals (adults) experiencing SMD, particularly in relation to oral health or other related health behaviours (e.g., substance misuse, smoking, diet) intervention or service. The sample will be drawn from Newcastle/Gateshead, London and Plymouth in order to obtain information on services and implementation approaches in different regions with high proportion of populations with SMD.

### 2.2. Sampling Strategy

Recruitment will be supported by an advisory group (which consists of independent and voluntary community sector organisations, NHS staff, lived experience representatives, and academics), existing research networks (e.g., Fuse mailing list), local research and development networks (e.g., clinical research networks), or primary care networks (e.g., the North of England Commissioning Support Unit), and other forms of opt-in email distribution lists. The recruitment of those with experience of SMD will be through our partner Fulfilling Lives and other organisations which provide services and support for people with experience of SMD. Posters, leaflets and word of mouth will be used to reach out to potential participants.

SMD population: Purposive sampling will be utilised to recruit participants. Sampling criteria for key stakeholders with experience of SMD will be: age, gender, homelessness, substance misuse and repeat offending. Participants will be offered a shopping voucher for taking part in an interview.

Service providers, commissioners, policy makers: Purposive sampling will also be utilised to recruit participants representing service providers and policy makers. Sampling criteria will be by organisation type (e.g., Local Authority, NHS England, Public Health England, voluntary sector) and by the types of support that are provided (e.g., focus on housing, offending, substance misuse, oral health) to ensure that there is sufficient representation from these various services that support SMD groups that are related to this project. Examples of roles of individuals who might participate include managers, commissioners, housing officers, community dentists, pharmacists, GPs, and support workers for substance misuse. Any staff from the NHS will most likely be dentists or nurses from NHS Community Dental Services and other relevant primary care staff who provide services for vulnerable populations, such as SMD groups.

### 2.3. Patient and Public Involvement (PPI)

In line with principles of co-production, input and engagement with individuals with lived experience of SMD will be sought in all stages of the study to ensure that the voice of this vulnerable population is represented. Individuals with experience of SMD who are trained and involved in research as “peer researchers” will be part of the study. Peer researchers have been involved in the planning of this study through our partners at Fulfilling Lives Newcastle/Gateshead (an organisation that supports people experiencing SMD). Input from peer researchers will be sought in the recruitment, data collection, data analysis, and dissemination stages. Peer researchers will be compensated for their time contributed to the study, which will be in the form of shopping vouchers. We have also had input from stakeholders which span various organisations that support SMD groups (e.g., Public Health England, charity organisations), and will seek their input in data collection and dissemination as the study progresses.

### 2.4. Data Analysis

All data will be audio recorded and transcribed, some by the research team and the rest by a transcription company. All identifiable data will be removed from the transcriptions by a member of the research team. Data will be analysed thematically, adopting an iterative approach based on the constant comparative method [15]. The constant comparative method will involve constantly comparing similarities and differences in information emerging from data, which will then be used to form categories and themes of findings. Analysis will focus on factors relating to the planning, adoption and feasibility of implementing interventions in SMD groups. A descriptive profile of the types of services/interventions, agencies/organisations involved will also be built. Qualitative software (NVivo) will aid in organising thematic codes and categories. Scrutiny and analysis of data will take place concurrently with data collection and iteratively. Discussions with peer researchers (individuals with lived experience of SMD) and the broader research team will be used to inform further data collection and analysis. Themes generated from the data will be reviewed at a stakeholder engagement workshop (broader PPI event) for input into policy implications and as evidence for the practice guide.

### 2.5. Ethical Approval and Considerations

This study has been as approved by the Faculty of Medical Sciences Research Ethics Committee, part of Newcastle University’s Research Ethics Committee (Ref: 9727/2020; 2066/9725).

### 2.6. Dissemination

A stakeholder engagement workshop will be held to reach consensus on recommendations and to conduct preliminary dissemination. The findings will be published in an appropriate peer-reviewed journal. The research findings will also be shared amongst our research partners and other networks. For example, members of our project who are embedded in local government, NHS, PHE and the third sector will facilitate the translation of our research into policy and practice, and disseminate the findings nationally. A key outcome of the study will be an ‘evidence for practice’ briefing document, which will be disseminated to relevant networks, including Commissioning and Health and Wellbeing Boards across the health and social care sector. The findings of the study will also be presented in a logic model. Other networks include Equal North Network, Policy Research Unit on Behavioural Science, and Fuse (Centre for Translational Research in Public Health). Furthermore, the study team will also look at ways in which the findings can be shared amongst SMD groups and associated organisations (e.g., through relevant newsletters).

## 3. Conclusions

People experiencing homelessness have a high burden of unmet needs in terms of poor oral health and related behaviours of substance use, smoking and poor diet. There is limited evidence on how interventions or services are developed and delivered in such a way that they aid acceptability and implementation amongst SMD populations, aiming to improve oral health and related behaviours. This study will draw on insights from key stakeholders, including people with experience of SMD and service providers, commissioners and policy makers, for developing such interventions. The findings of this study will provide information on aspects of interventions that could improve their implementation and acceptability for people experiencing SMD.
